# Identifying Allosteric Small-Molecule Binding Sites of Inactive NS2B-NS3 Proteases of Pathogenic *Flaviviridae*

**DOI:** 10.3390/v17010006

**Published:** 2024-12-24

**Authors:** Hovakim Grabski, Siranuysh Grabska, Ruben Abagyan

**Affiliations:** 1 Skaggs School of Pharmacy and Pharmaceutical Sciences, University of California, La Jolla, San Diego, CA 92093-0657, USA; sgrabska@health.ucsd.edu; 2 L.A. Orbeli Institute of Physiology, National Academy of Sciences, Yerevan 0028, Armenia

**Keywords:** Zika virus, protease inhibitors, mutation rates, allosteric druggable pockets, Dengue, Yellow Fever, Japanese encephalitis, NS2B, NS3

## Abstract

Dengue, West Nile, Zika, Yellow fever, and Japanese encephalitis viruses persist as significant global health threats. The development of new therapeutic strategies based on inhibiting essential viral enzymes or viral–host protein interactions is problematic due to the fast mutation rate and rapid emergence of drug resistance. This study focuses on the NS2B-NS3 protease as a promising target for antiviral drug development. Promising allosteric binding sites were identified in two conformationally distinct inactive states and characterized for five flaviviruses and four Dengue virus subtypes. Their shapes, druggability, inter-viral similarity, sequence variation, and susceptibility to drug-resistant mutations have been studied. Two identified allosteric inactive state pockets appear to be feasible alternatives to a larger closed pocket near the active site, and they can be targeted with specific drug-like small-molecule inhibitors. Virus-specific sequence and structure implications and the feasibility of multi-viral inhibitors are discussed.

## 1. Introduction

Finding small-molecule therapeutics to treat emerging flaviviral diseases, which are dangerous to human health, remains an unsolved problem. The most problematic mosquito-vectored *Flaviviridae* are the following: Zika virus (ZIKV), four subtypes of Dengue virus (DENV), West Nile virus (WNV), Japanese Encephalitis virus (JEV), and Yellow Fever virus (YFV) [1,2,3]. The existing supportive care and symptomatic treatments have limited efficacy, calling for the exploration of new viral targets essential for viral infectivity and growth, as well as identifying the relevant functional states for structure-based drug design [4]. These treatments should be able to reduce or prevent severe viscerotropic injuries caused by YFV, protect the human brain from the neurotropic injuries accompanying JEV, ZIKV, DENV, and WNV-related diseases [5], and prevent immune-mediated complications [6], cytokine storm, systemic vascular leakage, organ failure, or sepsis [7].

**Figure 1:**
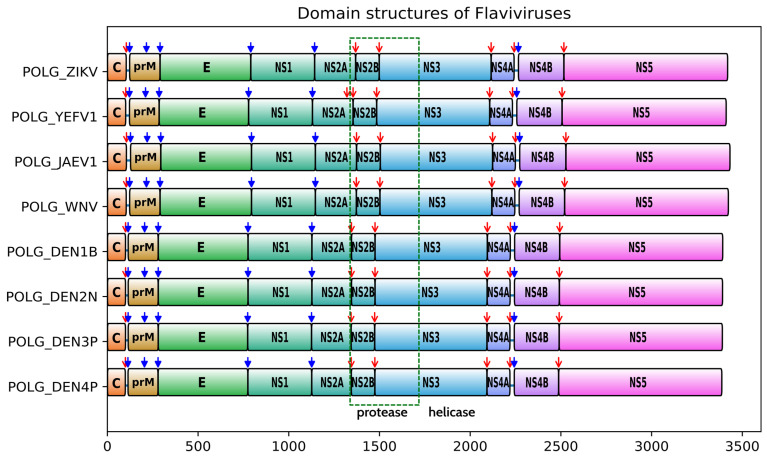
Domain organization of the polyprotein precursor encoded by different flaviviruses. Red arrows indicate the cleavage by the NS2B-NS3 protease, and blue arrows indicate the cleavage by the host cell proteases. A green box shows the two domains of the protease.

The genomes of the eight flaviviruses of interest consist of single-stranded positive-sense RNA coding for the polyprotein (PP) precursor [8,9]. The length and protein composition of the polyprotein are similar for the eight viruses [10,11,12,13,14,15]. The polyprotein consists of three structural proteins, capsid (C), the precursor membrane glycoprotein (prM), and the envelope glycoprotein (E), and seven nonstructural proteins (NS1, NS2A, NS2B, NS3, NS4A, NS4B, and NS5) [16,17,18,19,20] (Figure 1). The structural proteins are assembled in the endoplasmic reticulum and processed in the Golgi apparatus, where the viral particles mature into infectious virions. Non-structural proteins form replication complexes in the endoplasmic reticulum to replicate viral RNA [21]. The polyprotein is cleaved by the viral protease NS2B-NS3-pro and human proteases. This cleavage is essential for viral maturation and growth, thus making the NS2B-NS3 inhibition an attractive target for an antiviral agent [22,23,24].

The viral protease domain is designed in a particularly intricate manner. The active protease requires an NS2B fragment that wraps around the rest of the protease domain that includes the catalytic triad (His51, Asp75, and Ser135) [8,19]. NS3, a dual-function protein, consists of two domains: the N-terminal trypsin-like serine protease (NS3-pro) that binds to NS2B and the C-terminal helicase (NS3hel). NS2B acts as a cofactor for NS3-pro, undergoing a conformational change necessary to activate the protease. The multiple sequence alignment of NS2B-NS3-pro-NS3hel revealed a high level of sequence conservation [8,17,19,25]. However, it remains uncertain whether the inhibitor binding sites exhibit an even higher degree of conservation, warranting efforts to develop a single pan-flaviviral inhibitor.

The inhibition of the active site pockets of NS2B-NS3 protease has been studied recently as a therapeutic strategy [17,21,26,27]. Highly potent macrocyclic inhibitors of the Zika protease active site with Ki under 5 nM were identified [28]. However, the antiviral efficacy in cell-based assays was low, only in the micromolar range, according to a follow-up study from the same laboratory [29]. Therefore, a search for an alternative “inactive” conformational state and related allosteric pocket began. Our previous study identified compound R107 as a potential inhibitor of the “super-open” and inactive allosteric pocket of the NS2B-NS3-pro protease of ZIKV [24]. In the current study, we explored the feasibility of extending the initial finding for ZIKV to other infectious flaviviruses by analyzing over 13,600 sequences and 92 three-dimensional structures for the sequence variability of each virus in the vicinities of three identified pockets. The side chains lining the pockets of all proteases were studied for both intra-species variations and inter-species differences for the five viruses and three additional Dengue types. The impact of that variation on drug resistance, the “druggability” of the allosteric pockets, and the feasibility of small-molecule pan-viral inhibitors are presented.

## 2. Materials and Methods

### 2.1. Collection, Superposition, and Analysis of 3D Structures of NS2B-NS3 Proteases

The NS3-pro domain sequences extracted from the flavivirus sequences were searched against the Protein Data Bank, PDB [30], to identify close sequence matches with experimentally determined protein structures by NCBI Blast with a threshold of 90% sequence identity. The structures were split into individual conformationally different conformers of NS2B-NS3 and superimposed by NS3 to further classify them into the three conformational and functional states: closed active state, transient inactive state, and a fully opened inactive state (also known as “super-open”). The initial preparation has been performed as follows:

For ZIKV, 59 structures were used for analysis. These structures contained NS2B and NS3-pro subunits. Most of those structures had an NS2B chain as a separate polypeptide cleaved from the NS3 domain, while some contained one continuous NS2B-NS3 polypeptide chain. We assigned uniform residue numbers for residues of each domain.

Three structures available for YFV were kept as is. For WNV, six structures were analyzed. One JEV protease structure, 4R8T [31], was used.

DENV-1 was represented by 3L6P [32] and 3LKW [32] entries, each containing a single chain with both protease domains. For DENV-2, eleven protease structures were retained. For Dengue 3, two structures, 3U1I [33] and 3U1J [33], were identified. Finally, ten DENV-4 structures were used for this study.

### 2.2. Sequence Analysis

The deposited RNA sequences of all studied viruses have been downloaded from the NCBI Virus community portal that combined RefSeq, GenBank, and other NCBI repositories [34]. In total, 13,692 sequences were used for the pocket conservation analysis.

#### 2.2.1. Zika Virus NS2B-NS3 Sequence Set Compilation and Alignment

The ZIKV set contained 2483 polyprotein amino acid sequences. They were preprocessed to extract only the NS2B and NS3-pro regions (residues 1369–1676 according to the InterPro entry PS51527). Moreover, 980 protease sequences (82 unique sequences) fully representing the set have been aligned using the zero-gap global alignment algorithm ZEGA within ICM-PRO v3.9-3b [35]. The alignment was analyzed for position-dependent variability and attribution to three different pockets according to a 3D model of NS2B-NS3-pro.

#### 2.2.2. West Nile Virus NS2B-NS3 Sequence Set Compilation and Alignment

The WNV set contained 7627 polyprotein amino acid sequences. They were preprocessed to extract only the NS2B and NS3-pro regions (residues 1371–1502 for NS2B and 1502–1679 for NS3-pro according to the InterPro [36] entry PS51527). Moreover, 486 protease sequences (67 unique sequences) fully representing the set have been aligned using the zero-gap global alignment algorithm ZEGA within ICM-PRO v3.9-3b [35]. The alignment was analyzed for position-dependent variability and attribution to three different pockets according to a 3D model of NS2B-NS3-pro.

#### 2.2.3. Yellow Fever Virus NS2B-NS3 Sequence Set Compilation and Alignment

The YFV set contained 1967 polyprotein amino acid sequences. They were preprocessed to extract only the NS2B and NS3-pro regions (residues 1371–1665 according to the InterPro [36] entry PS51527). Moreover, 910 protease sequences (119 unique sequences) fully representing the set have been aligned using the zero-gap global alignment algorithm ZEGA within ICM-PRO v3.9-3b [35]. The alignment was analyzed for position-dependent variability and attribution to three different pockets according to a 3D model of NS2B-NS3-pro.

#### 2.2.4. Japanese Encephalitis Virus NS2B-NS3 Sequence Set Compilation and Alignment

The JEV set contained 3292 polyprotein amino acid sequences. They were preprocessed to extract only the NS2B and NS3-pro regions (residues 1374–1504 for NS2B and 1505–1682 for NS3-pro according to the InterPro [36] entry PS51527). Moreover, 246 protease sequences (53 unique sequences) fully representing the set have been aligned using the zero-gap global alignment algorithm ZEGA within ICM-PRO v3.9-3b [35]. The alignment was analyzed for position-dependent variability and attribution to three different pockets according to a 3D model of NS2B-NS3-pro.

#### 2.2.5. Dengue-1, -2, -3, and -4 Virus NS2B-NS3 Sequence Set Compilation and Alignment

The DENVs set contained 15,387 for DENV-1, 13,326 for DENV-2, 7126 for DENV-3, and 3889 for DENV-4 polyprotein amino acid sequences. They were preprocessed to extract only the NS2B and NS3-pro regions (residues 1346–1475 for NS2B and 1476–1653 for NS3-pro according to the InterPro [36] entry PS51527). The fully represented sequence set has been aligned using the zero-gap global alignment algorithm ZEGA within ICM-PRO v3.9-3b [35]:

DENV-1: 4628 sequences (614 unique sequences)
DENV-2: 3816 sequences (553 unique sequences)
DENV-3: 1739 sequences (263 unique sequences)
DENV-4: 862 sequences (202 unique sequences)

The alignment was analyzed for position-dependent variability and attribution to three different pockets according to a 3D model of NS2B-NS3-pro.

### 2.3. Analysis and Visualization

The analysis, superposition, and visualization were performed using ICM-Pro 3.9-3b molecular modeling software [35,37,38]. The domain plots of the polyprotein precursor were generated with Python and matplotlib [39] based on the data from InterPro and UniProt [36,40]. The assignment of the three functional and conformational states to each protease structure was carried out as follows:

- The active *closed* state is defined by the distance between Cα-atoms of D75 of NS3-pro and G82 of NS2B being smaller than 10 Å. All other models of the inactive state with the NS2B loop unwrapped are further sub-divided by the conformation of the C-terminal end of NS3-pro according to the values of the angle defined below.
- The inactive *transient* state is characterized by the C-terminal fragment hairpin displacement following the unwrapping of NS2B. The extension of the C-terminal end region for this state is defined by the Cα atoms of G148, N152, and S158 (numbering from DENV-2) residues of NS3-pro being over 90°.
- The inactive *fully opened* state is characterized by the hairpin unfolding and rearrangement of the NS3-pro C-terminal fragment, with the angle defined above being less than 90°.

## 3. Results

To analyze drug-targetable allosteric binding sites in the inactive states of the protease, we started by analyzing all experimentally determined structures of the proteases of five flaviviruses, introducing a uniform NS2B-NS3-pro numbering for each virus type and subtype, classifying them by the conformations of NS2B and the NS3-pro C-terminal fragment relative to the protease core, and assigning each protease one of three conformational states: closed, transient, and fully opened. Finally, new druggable pockets in the transient and fully opened state were predicted, and sequence variability of immediate pocket surfaces was analyzed. A total of 92 models for five flaviviruses, three subtypes, in up to three conformational states have been collected and superimposed, and 13,692 sequences of the NS2B-NS3 proteases analyzed.

### 3.1. Three Targetable Conformational States of NS2B-NS3-Pro for the Five Viruses

**Figure 2:**
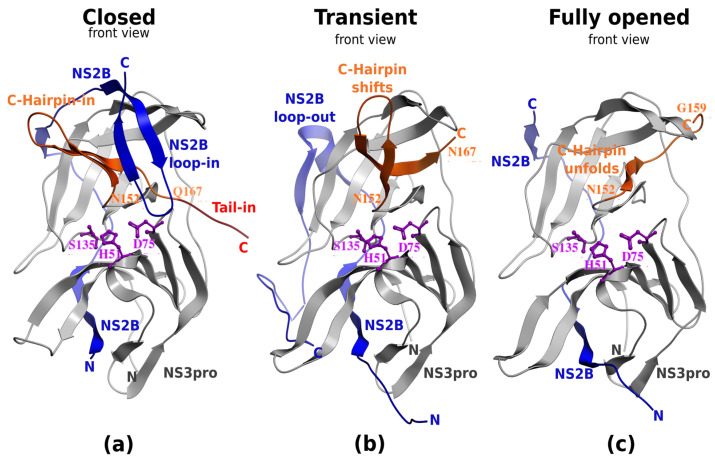
The mobile structural determinants of the three states in existing crystallographic structures of NS2B-NS3 protease domains are shown by blue and orange backbone ribbons. The closed state (**a**) is exemplified by PDB ID 5YOF, the transient state (**b**) by 2FOM, and the fully opened (**c**) by 7M1V. The backbone ribbon is colored as follows: blue ribbon—mobile NS2B, gray ribbon—static NS3 core, orange ribbon—mobile NS3-pro-C-terminal hairpin. The catalytic triad is shown and labeled for reference.

Ninety-two PDB entries for ZIKV, WNV, YFV, JEV, DENV-1, -2, -3, and -4 (eight groups in total) were analyzed. Each of the entries was processed to extract the best single representative of the NS2B-NS3-pro domain, superimposed to a common reference conformation of the core NS3-pro domain, and assigned one of three conformational states, one active state, and two inactive ones (Figure 2). The geometrical definitions of the conformational states defined by the NS2B and C-hairpin of NS3-pro are described in Section 2.3. Each of the eight viruses had PDB structures in at least one of the three states.

Most structures (69 out of 92) represented the closed/active state, and most co-crystallized inhibitors were bound in the close vicinity of the active site (Table 1). Most closed-state structures were determined for ZIKV protease (53). The other three viruses and four Dengue subtypes had the following number of structures of the closed state: WNV (5), YFV (1), JEV (0), DENV-1 (0), DENV-2 (2), DENV-3 (2), and DENV-4 (6). We used those structures to predict allosteric binding sites of the closed state (see Section 3.3).

The inactive transient state was identified in 16 structures out of 92. All those structures were analyzed for alternative allosteric binding pockets that may be targeted for stabilizing the inactive state and/or interfering with the substrate binding. Those pockets, if identified, may represent promising sites for drug design.

Seven structures exhibited a fully opened inactive state with NS2B fully unwrapped and the C-terminal hairpin unfolded and extended. Interestingly, this C-terminal extension creates a new cavity that may be targeted by a small molecule. The fully opened state conformational was found in six ZIKV PDBs and one JEV structure. The predicted allosteric pockets in inactive conformations overlapped with the pockets identified in the transient states but were different from them. Given the variable nature of the inactive states and the related allosteric pockets, these additional conformations and pockets presented alternative inactive-state pockets for ligand screening.

To deduce the “druggability” of three different types of allosteric pockets, we analyzed the known co-crystallized ligands. Table 1 summarizes the distribution of the conformational states of the proteases for the five viruses and Dengue subtypes with and without the co-crystallized ligands.

#### 3.1.1. Closed Conformation/Active State

Most of the closed conformations with the active site pocket (ASP) were solved with a peptide-like inhibitor. A smaller number of closed structures were crystallized with small-molecule ligands only for the Zika virus; however, all the ligands were notably weak binders. The affinities span a micromolar range of 1.5 mM to 200 mM [41,42,43,44] (PDB: 5YOD, 5H4I, 7VVX, 6L4Z, and 6L50); e.g., the 5YOD [41] ligand has an IC50 of 1.5 mM, the 5H4I [42] ligand has an IC50 of 14.08 mM, the 7VVX [43] ligand has an IC50 of 48.7 mM, and the 6L4Z [44] and 6L50 [44] were co-crystallized with ligands of IC50 of 200 mM. These values indicate the limitations associated with targeting the active site pocket of the Zika protease and likely for the other viruses given the ASP similarities.

Structural reasons making the ASP difficult for targeting with small molecules include its charge and shape properties, namely three negatively charged residues, two aspartic acids in NS3-pro, and one glutamic acid in NS2B for YFV, DENV-1, -2, and -4 [41]. In addition, the pocket is relatively flat. While the research aimed at the identification of both covalent and non-covalent inhibitors with promising therapeutic potential continues [45,46,47,48,49], we focused on identifying a different location for a small-molecule inhibitor.

#### 3.1.2. Open Conformation/Inactive State

The two structures in a fully opened conformation were crystallized in an inactive apo-state and with a weak (IC50 over 1 µM) small-molecule inhibitor (PDB: 7M1V), both for the Zika protease [25]. This allosteric pocket, distant from the active site, was recently targeted with a structure-based docking screen and experimental testing, and a weak allosteric inhibitor was identified [24]. However, this promising binding site can be targeted for more potent and selective inhibitors.

Each of the DENV-1, -2, and -4 subtypes of the Dengue virus protease had at least one transient or fully opened state structure determined experimentally (Table 1). Notably, three DENV-2 transient state structures, 6MO0, 6MO1, and 6MO2 [50], were co-crystallized with IC50 from 200 to 860 nM small-molecule inhibitors [50], useful for both the pocket definition and attempts to improve the inhibition efficacy. The distinction between the two inactive states for these three structures was challenging because the C-terminal hairpin residues were missing; we used the visible stem strands of the loop for the assignment to the transient state. These inhibitors helped to define an allosteric pocket for all Dengue viruses. This ligand-defined pocket is different from the ligand-defined ZIKV pocket [24,51] even though the two pockets overlapped partially.

The inactive state proteases were crystallized for other flaviviruses, but the structural information is insufficient and completely missing for YFV. Only an apo-structure in the inactive transient conformation (2GGV) [19] was determined for the West Nile virus protease, and one fully opened inactive structure of JEV has been solved (4R8T) [31].

In summary, there is sufficient structural information for targeting allosteric pockets in one of the two inactive states with small molecules as a strategy for developing novel small-molecule inhibitors against all five flaviviruses studied. For proteases with multiple structures in the inactive state, we analyzed the structural variation of each protease in order to be able to evaluate more and less conformationally defined surfaces of the protease and to select the most suitable locations and representatives for further docking screens.

### 3.2. Conformational Variations Among Flaviviral NS2B-NS3 Proteases Across the Three States

For comparative analysis, representative structures for each of the five viruses and Dengue-1, -2, -3, and -4 subtypes were selected with a preference for better resolution and fewer residues missing from the model, or zero occupancy for each of the three states. The following models were prepared and converted to full atom models: the closed state: ZIKV (5YOF) [41], WNV (2FP7) [52], YFV (6URV) [53], DENV-2 (2M9P) [54], DENV-3 (3U1I) [33], and DENV-4 (5YW1) [55]; the transient state: WNV (2GGV) [19], DENV-1 (3L6P) [32], DENV-2 (2FOM) [52], and DENV-4 (2VBC) [56]; and the fully opened state: ZIKV (7M1V) [25] and JEV (4R8T) [31]. The closed active state in this analysis was included for comparison with the two inactive states.

**Figure 3:**
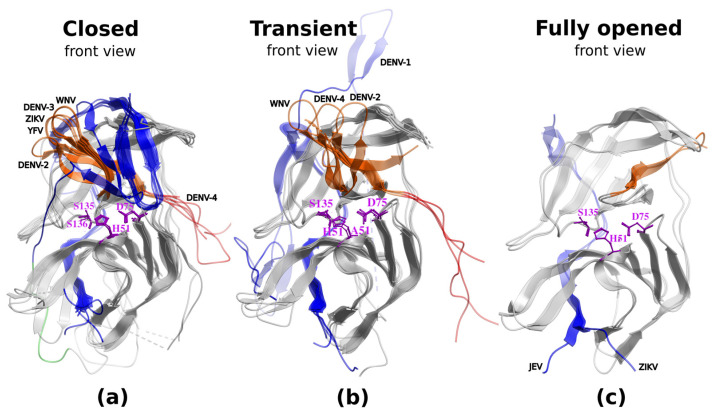
Structural variations within each of the closed (**a**), transient (**b**), and fully opened (**c**) states of the flaviviral NS2B-NS3-pro domains for all five flaviviruses and four Dengue subtypes under study. Some differences in residue numbers (YFV S136 instead of S135 for other viruses) are due to sequence length variations in both NS2B and NS3-pro subunits (**a**). The NS2B loop in one of the closed-state DENV-2 constructs was conformationally shifted due to an inserted linker in a crystallized construct (see a green backbone fragment in (**a**)).

The active state conformations of ZIKV, WNV, YFV, DENV-3, and -4 reveal that the closed conformation is structurally conserved not only inside each of the Dengue subtypes but also across the five species (Figure 3a). The catalytic triad is aligned perfectly between the viruses.

The calculation of the backbone deviations of the active site vicinity of the closed state, including the NS2B and C-terminal fragment, shows the conformational conservation of the active site vicinity between the viruses and subtypes (Table 2). The transient state characterized by the NS2B unwrapping and NS3-hairpin preservation shows the hairpin, forming a complete loop directed upwards. Furthermore, its absence in several flavivirus crystallographic structures (6MO0-2, 2WHX [57], 2WZQ [57], and 5YVJ [58]) confirms this flexibility. The NS2B loop rotates and extends backward rather than closing the upper part of the active site. This flexibility creates ample space for the NS3-loop to adopt varying conformations across different flaviviruses, as observed in crystallographic structures from WNV, DENV-1, -2, and -4. In the transient conformation of DENV-1, the NS2B exhibits a distinct conformation different from other flaviviruses and is directed upwards due to binding to crystallographic neighbors (PDB IDs: 3L6P and 3LKW).

Although there are fewer examples of inactive complexes for transient and fully opened conformations, those conformations are sufficiently conserved to explore alternative mechanisms of inhibition of flavivirus proteases by stabilizing the inactive state. The most promising state for stronger small-molecule binders is the transient state, despite some variability of the C-terminal fragment of NS3-pro.

### 3.3. Identification of Druggable Pockets in Two Inactive States of the NS2B-NS3 Protease

To identify possible druggable pockets, we performed pocket prediction using ICMPocketFinder [59]. ICM pocket finder relies on generating cumulative grid-potential-based maps that evaluate van der Waals interactions between the receptor and a virtual ligand and contouring the maps to identify contiguous density regions. Detailed characteristics of the active site and further evaluation of their druggability are performed with the DLID measure [60]. Three pocket locations, denoted as allosteric pockets 1, 2, and 3 (AP1, AP2, and AP3), were further analyzed for druggability and sequence conservation *between* the viruses and Dengue subtypes. The intra-viral sequence conservation around those pockets for each of the eight groups was analyzed in Section 3.4.

#### 3.3.1. Pocket Locations and Their Composition

**Figure 4:**
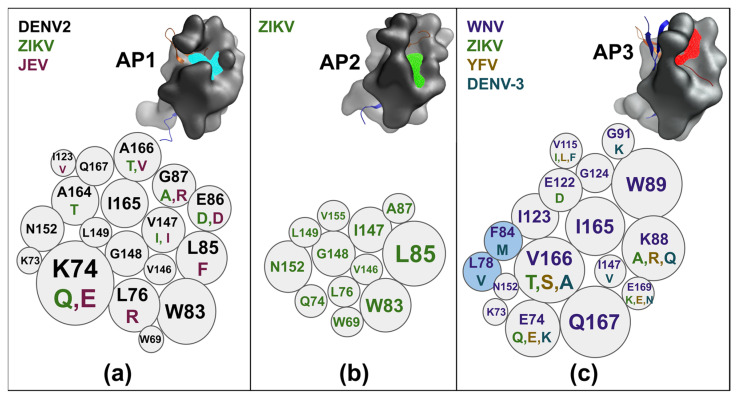
Allosteric pockets (AP1, AP2, and AP3) on flavivirus NS2B-NS3-pro proteins (gray surfaces) highlight conserved and variable residues. AP1 (**a**): Residues across DENV2 (black), ZIKV (green), and JEV (magenta) include highly variable sites like K74, E86, and L85. AP2 (**b**): Conserved residues in ZIKV (green) include L85, with surrounding residues contributing to structural integrity. AP3 (**c**): Variability across WNV (purple), ZIKV (green), YFV (dark yellow), and DENV3 (dark cyan) is shown, with key residues like W89, V166, and Q167. Bubble sizes reflect residue contribution to the pocket surface, with colors denoting specific viruses.

The three allosteric pockets were identified by the following procedure. First, the ICM pocket finder tool generated the likely pocket shapes, then we analyzed the overlap of those shapes with the co-crystallized ligands, analyzed for a consistent location in different virtual protease structures, and translated into a particular surface in terms of amino acid positions (Figure 4). The pockets are shown below, and the intra-viral conservation is shown by alternative amino acid codes in Figure 4.

The AP1 pocket within NS3-pro (Figure 4) was the pocket we (R.A.) screened to identify an allosteric inhibitor [24]. AP1 is formed by 18 amino acids, combining hydrophobic and polar features. Nine of those amino acids are conserved between three viruses with X-ray structures, and the other nine amino acids differ in ZIKV and JEV, thus changing the shape of AP1. Hydrophobic residues, such as L76 (R in JEV), W83, and V147, create a patch that can be targeted to increase the binding affinity of a tentative inhibitor. The polar residues, including K74 and E86 in Dengue-2, are different in ZIKV and JEV. The AP2 pocket (Figure 4) overlaps with AP1 and is only visible for ZIKV co-crystallized with a binder (PDB: 7M1V). Eleven out of twelve amino acids forming the pocket are common with AP1, but the shape is somewhat different, which may be essential in a structure-based docking screen.

The AP3 pocket has not been characterized previously, but it is clearly visible in structure analysis and pocket prediction in at least four viruses: ZIKV, WNV, Dengue-3, and YFV. The amino acid composition of AP3 is different and overlaps with AP1 for only seven amino acids out of 17. Several hydrophobic residues, including W89, I165, and I123, are well conserved and can be exploited for pan-flaviviral inhibitors. A large-scale screen for AP3 binders is in progress.

#### 3.3.2. Backbone Conformational Conservation of the Pockets Between Flaviviruses

In addition to the sequence variation around the active site pocket, AP1 and AP2, we further studied the conformational variation of the backbone surrounding the pockets to evaluate the prospect of identifying pan-flaviviral protease inhibitors.

For each pair of protease representatives of each virus and four Dengue protease states, we calculated the root mean square deviation (RMSD) measure of the pocket vicinity (Table 2). The NS2B-NS3 protease of DENV-2 exhibits the largest deviations among the proteases studied, potentially shedding light on the challenges of effectively targeting DENV-2 through binders.

In contrast, the pockets of ZIKV, WNV, and YFV proteases show smaller structural deviations. Some variations may be attributed to a designed construct and crystal conditions of a particular model; other variations suggest a separate structure-based screen for the deviated conformations of the pocket.

The predicted AP3 pocket overlaps with AP1 substantially with the exception of the distal end of the AP3 pocket proximal to the NS3-pro C-terminal hairpin. Therefore, the conformational conservation of the AP1 and AP3 backbones is similar.

The X-ray structures of NS3-protease may also be analyzed for average B-factors of residues lining the AP1 and AP2 pockets. For each of the PDB entries studied, we derived an average B-factor and separately calculated the mean B-factor of residues covering the pockets. The ratios of the pocket residue B-factors to the protein-wide mean B-factor value indicated the relative mobility of the binding site residues in each pocket and in each virus. Let us describe a few structures chosen as the best pocket representatives. The average *relative* B-factors of residues in the AP1 pocket were as low as 0.80 for DENV2 (6MO0, ligand-bound state), which means that the pocket is more structurally conserved compared to an average B-factor for all protein residues. The same relative AP1 3D-conservation number for DENV2 (2FOM, apo-state) was 1.28 when compared to the average protein atom B-factor. This number is higher than 1. or 0.9 because the 2FOM structure is in an unliganded apo-state. That results in less-ordered residues in the pocket. The average *relative* B-factors of residues in the AP2 pocket were as low as 0.88 for ZIKV (7M1V, ligand-bound state), which means that the pocket is more structurally conserved compared to an average B-factor for all protein residues. Overall, the pocket residues in the apo-state are comparable to, or more structurally conserved than, the surface residues and only slightly less ordered than the buried amino acids.

### 3.4. Sequential Variations Around Pockets and the Risk of Drug Resistance

**Figure 5:**
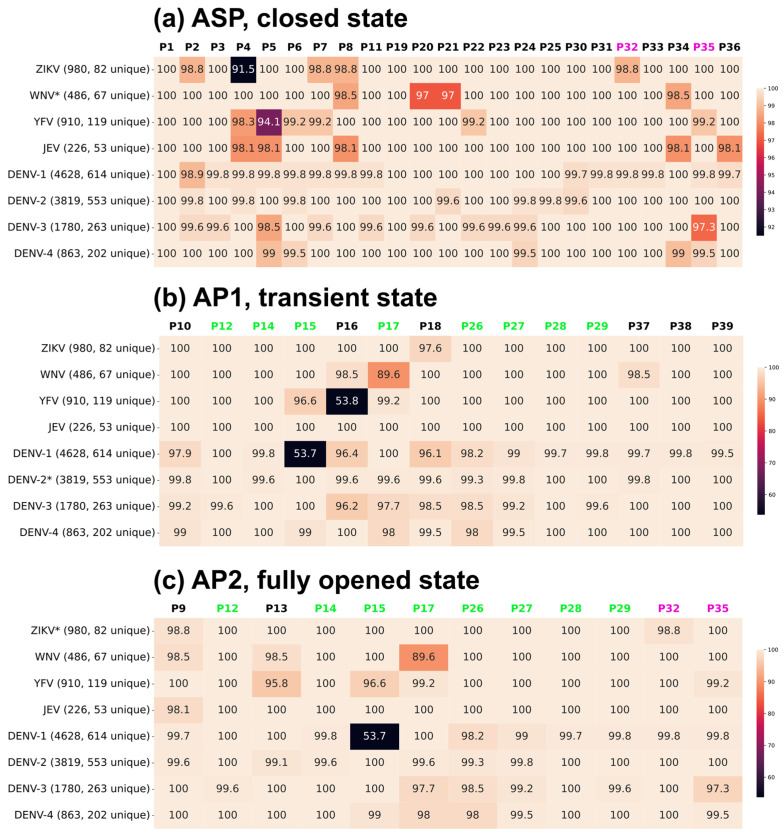
Sequence conservation (%) analysis of (**a**) active site pocket (closed state), (**b**) allosteric pocket 1 (transient), and (**c**) allosteric pocket 2 (fully opened state) within various flaviviruses. The letter “P” denotes the amino acid position within the pocket. The asterisk indicates the flaviviruses used to define the amino acids in the three pockets (closed—5IDK (WNV), transient—6MO2 (DENV-2), and fully opened—7M1V (ZIKV)).

Sequence conservation of amino acids for each of the five flaviviruses and additional Dengue subtypes can be derived from sequence data deposited to the NCBI Virus database. We were particularly interested in the relative conservation of the amino acids proximal to the three main targetable pockets. This variation needs to be taken into consideration to avoid sequence-dependent resistance to inhibitors. We analyzed the sequence conservation and diversity of the polyprotein precursor within and across five viruses and three additional Dengue subtypes (Figure 5) for the pocket vicinities, ASP, AP1, and AP2.

We retrieved a large number of amino acid sequences from the NCBI Virus database: 980 for ZIKV, 486 for WNV, 910 for YFV, 226 for JEV, 4628 for DENV-1, 3819 for DENV-2, 1780 for DENV-3, and 863 for DENV-4. The sequences were processed to extract the NS2B-NS3-pro fragments and further filtered to identify unique sequences. The next step was to extract amino acids in positions proximal to each of the three pockets studied.

For each of the conformational states, amino acids forming the pockets ASP, AP1, and AP2 were refined to subsets neighboring the co-crystallized ligands. A comparison of the amino acids forming ASP in the closed state across flaviviruses reveals that WNV’s pocket is the most conserved, with variation observed at only four positions. In contrast, DENV-1 has the most variable pocket. Despite the significant conservation of ASP in ZIKV, the largest mutation rate is observed at position ASP-P4, making this site prone to mutations. Similarly, a low conservation percentage was found in YFV at position ASP-P5. These sites should be considered and, if necessary, avoided during inhibitor design.

In the transient state, the most conserved AP1 is observed in JEV, with 100% conservation for all residue positions, while the least conserved ones are found in DENV-1 and -3. The conserved surface of the AP1 pocket in JEV makes it a good target.

AP2 was identified in the fully opened conformation. The most conserved AP2 was found for JEV, with mutability at only one position, while DENV-1-AP2 exhibits the most overall variability. In all three states, DENV-1-AP2 exhibited the highest variability, likely due to the availability of a larger number of amino acid sequences. In the transient state, the most variable sites include YFV at position 16, where D is present in 53.8% of sequences and E in 46.2%, and DENV-1 at position 15, with F in 53.7% and L in 46.3%. High variability is also observed at position P17 in WNV (89.6%) and at position P16 in YFV (53.8%). Those positions also need to be considered and avoided during drug development. Despite JEV and DENV-4 having the highest percentage of unique sequences, indicating their lower amino acid sequence conservation within the species, these viruses exhibit relative stability and sequence conservation in all three pockets, especially for JEV (Figure 5).

**Figure 6:**
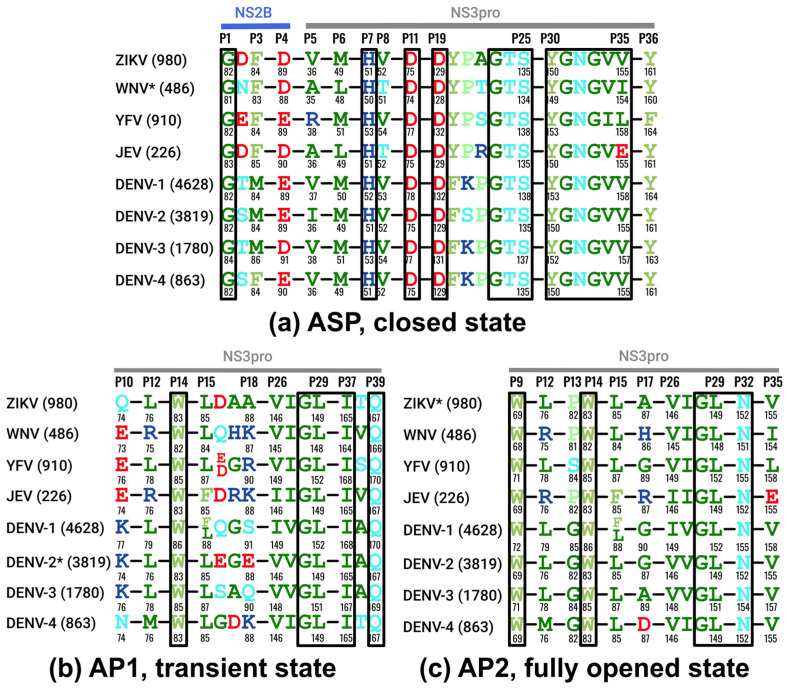
Amino acid sequence conservation analysis of (**a**) active site pocket (closed state), (**b**) allosteric pocket 1 (transient), and (**c**) allosteric pocket 2 (fully opened state) among various flaviviruses. The letter “P” denotes the amino acid position within the pocket. The asterisk indicates the flaviviruses used to define the amino acids in the three pockets (closed—5IDK (WNV), transient—6MO2 (DENV-2), and fully opened—7M1V (ZIKV)). To simplify the analysis, we focused on these specific flaviviruses as representatives for the determination of amino acid residues within the pockets. Amino acids identical among all flaviviruses are outlined in black rectangles. Each flavivirus has its own local numbering for NS2B and NS3.

We also compared sequences and subsets around the pockets of the most frequent amino acids for each of the viruses to evaluate a potential for multi-viral or even pan-viral inhibitors. Figure 6 shows pocket surroundings for ZIKV, WNV, YFV, JEV, and DENV-1 through DENV-4. The number of positions with identical amino acids in different viruses is only 47.83% around ASP, 35.71% for AP1, and 41.67% for AP2 (Figure 6).

Hence, developing drugs that target all flaviviruses simultaneously is a challenging task. For the ASP, it would mean avoiding positions P3–P6 and P20–P22. However, it is quite possible to develop a drug that targets one of the three pockets in more than one flavivirus. A drug could be developed to bind to the ASP in both ZIKV and YFV. It is also feasible to find a compound that would inhibit the closed state of five flaviviruses at once: WNV and the four DENV serotypes.

Targeting AP1 in the transient state with a drug effective across multiple flaviviruses presents a significant challenge. However, DENV-1 and DENV-3 would be good candidates for a single AP1-binding inhibitor targeting two subtypes. Inhibiting allosteric pocket 2 in the fully opened state across multiple flaviviruses appears to be attractive for simultaneous inhibition of DENV-1, -2, and -3 because all positions around AP2 are identical or similar (G-P17-A) between the three Dengue subtypes. DENV-4 AP2 pocket residues include two additional mutations, with P17:G-to-D being a more significant change.

In summary, the AP1 is the most promising pocket for targeting, even though it may be difficult to find a pan-flaviviral NS3-pro inhibitor with the same efficacy.

## 4. Discussion

The global impact of flaviviruses remains substantial, affecting hundreds of millions of people each year. This underscores the urgent need for effective antiviral therapies. Developing selective small-molecule compounds capable of targeting and inhibiting flaviviruses is a critical priority. Among potential therapeutic targets, the NS2B-NS3 protease complex stands out due to its pivotal role in viral replication, making it a highly promising focus for anti-flaviviral drug development. Unfortunately, no approved anti-flaviviral drugs are currently available, prompting ongoing research efforts to identify selective and potent inhibitors for NS2B-NS3-pro proteases of the flaviviruses [18,24,46,47,61].

Here, we studied NS2B-NS3-pro protease activity-specific allosteric binding sites in ZIKV, WNV, YFV, JEV, and DENV-1, -2, -3, and 4 proteases in three distinct conformational states, i.e., closed, transient, and fully opened identified previously [25,44,53,56,62,63,64]. Our main objective was to identify and characterize the binding sites alternative to the pocket near the closed active site pocket. The main reason for that search was that the active site inhibitors identified so far were not sufficiently potent [41,42,65,66]. Among several obstacles, three negatively charged amino acids around ASP (Figure 6) create a total negative charge [41] at the pocket, and the pocket shape is relatively open. Nevertheless, research is still ongoing to search for covalent (S135) or non-covalent inhibitors [45,46,47,48].

On the other hand, the open state allosteric pockets, AP1 [24] and AP2 pockets, provide a better opportunity, as Figure 6 shows; their surface is not charged, and the shape is more enclosed. We followed the definitions of the allosteric pocket as formulated in [67] in a sense that the allosteric pockets were sufficiently close to the active site and the substrate binding site but distinct from it. In addition, we had preliminary evidence that some of the allosteric site ligands identified so far inhibited the protease activity by either stabilizing the inactive state or directly interfering with the substrate binding. Some inhibitors against those open state pockets have been found: AP1 [24,26] and AP2 [51]. Both allosteric pockets are primarily hydrophobic, with AP2 being even more hydrophobic than AP1. For the AP1 in the transient state, there are crystallographic structures with small-molecule compounds [50] for DENV-2. However, not every structure of the transient state was suitable for structure-based docking screens due to the missing fragments around the AP2 pocket [68]. Overall, the AP1 pocket models in the transient state can be found in the PDB for three flaviviruses (DENV2, ZIKV, and JEV) already and can be modeled for the remaining two viruses and subtypes. AP2 is a less investigated pocket [25] because it is visible only in one crystal structure in Zika in a fully open state.

Finally, the AP3 is the only pocket that is allosteric, yet it is present in both the closed active state and in the transient state. The AP3 pocket is the least studied one yet is promising in terms of its composition and shape in comparison with the active site pocket.

The sequence conservation within each species (intraspecies analysis) revealed that two allosteric pockets of the inactive state for all viruses are well conserved for targeting (Figure 5), with JEV AP1 surface being the most conserved (100%) among the flaviviruses. The backbone conformation around the AP1 and AP2 pockets was also conserved sufficiently between different viral proteases, with one notable exception of the AP1 pocket of the Dengue-2 structure (RMSD to other subtypes and viruses close to 3A) in which a C-terminal fragment of NS3-pro moves upward. With this caveat, the AP1 pocket appears to be a prime target for structure-based screening for potential inhibitors against large compound databases or peptides and peptidomimetics.

While targeting multiple flaviviruses simultaneously is a challenging task, it is not impossible if inhibitor interactions are focused on the conserved pocket patches only.

## 5. Conclusions

Targeting the NS2B-NS3 protease of flaviviruses remains a promising antiviral strategy due to its critical role in viral replication. However, targeting the active site of the closed state of the protease proved to be challenging. Our study explored allosteric binding sites, focusing on two distinct conformational states of the inactive protease—transient and fully opened—and compared them with the closed (active) state. The two allosteric pockets in the inactive states emerged as “druggable” due to their hydrophobic nature and enclosed shape, with a transient state pocket showing notable sequence conservation across flaviviruses. Our analysis of both intra-viral and inter-viral variations of pocket sequences and 3D conformations revealed that virus-specific allosteric inhibitors can achieve sufficient efficacy by structure-guided improvements. Continued exploration of these sites and structure-guided screening of those pockets against small-molecule libraries or peptides/peptidomimetics hold promise for advancing anti-flaviviral drug discovery.

## Figures and Tables

**Table 1:** Comparative analysis of the existing crystallographic structures of NS2B-NS3 in relation to their conformational states. The sorting of the crystallographic structures of NS2B-NS3 into three conformational states was conducted based on criteria described in Section 2.3.

Flaviviruses	Closed			Transient		Fully Opened	
Abbreviation	*With a Peptide-like Inhibitor*	*With a Ligand*	*Without a Ligand*	*With Small-Molecule Inhibitor*	*Without a Ligand*	*With Small-Molecule Inhibitor*	*Without a Ligand*
ZIKV	5LC0, 5GJ4, 5H6V, 5YOF, 5ZMQ, 5ZMS, 5ZOB, 6JPW, 6KK2, 6KK3, 6KK4, 6KK5, 6KK6, 6KPQ, 6Y3B, 7DOC, 7O2M, 7O55, 7OBV, 7OC2, 7PFQ, 7PFY, 7PFZ, 7PG1, 7PGC, 7VLG, 7VLH, 7VLI, 7VXY, 7ZLC, 7ZLD, 7ZMI, 7ZNO, 7ZPD, 7ZQ1, 7ZQF, 7ZTM, 7ZUM, 7ZV4, 7ZVV, 7ZW5, 7ZWK, 7ZYS, 8A15, 8AQA, 8AQB, 8AQK	5H4I, 5YOD, 6L4Z, 6L50, 7VXX	5GPI			7M1V	5GXJ, 5T1V, 5TFN, 5TFO, 6UM3
WNV	2FP7, 2IJO *, 2YOL, 3E90, 5IDK				2GGV		
YFV			6URV				
JEV							4R8T
DENV-1					3L6P, 3LKW		
DENV-2	2M9P, 2M9Q			6MO0, 6MO1, 6MO2	2FOM, 4M9F, 4M9I, 4M9K, 4M9M, 4M9T		
DENV-3	3U1I, 3U1J *						
DENV-4	5YVU *, 5YW1 *		5YVV, 5YVW, 5YVY, 7VMV		2VBC, 2WHX, 2WZQ, 5YVJ		

* Crystallographic structure of NS2B-NS3 with bovine pancreatic trypsin inhibitor.

**Table 2:** Root mean square deviation (RMSD, in Å units) calculations of the backbone atoms between residues forming the ASP in the closed/active state of ZIKV, WNV, YFV, DENV-2, DENV-3, and DENV-4 flaviviruses; the AP1 in the transient state (WNV, DENV-1, DENV-2, and DENV-4); and the AP2 in the fully opened state (ZIKV and JEV).

	**ASP, CLOSED STATE**					
	**ZIKV**	**WNV**	**YFV**	**DENV-2**	**DENV-3**	**DENV-4**
**DENV-4**	0.9	0.7	0.7	3.7	**0.5**	0.0
**DENV-3**	0.8	0.6	0.7	3.97	0.0	
**DENV-2**	3.8	3.99	3.9	0.0		
**YFV**	0.5	0.6	0.0			
**WNV**	**0.4**	0.0				
**ZIKV**	0.0					
	**AP1, TRANSIENT STATE**					
	**WNV**	**DENV-1**	**DENV-2**	**DENV-4**		
**DENV-4**	0.7	0.7	2.9	0.0		
**DENV-2**	2.97	2.9	0.0			
**DENV-1**	0.7	0.0				
**WNV**	0.0					
**AP2, FULLY OPENED STATE**						
	**ZIKV**	**JEV**				
**JEV**	0.7	0.0				
**ZIKV**	0.0					

## Data Availability

Data can be obtained by contacting the respective authors.
